# The Independent Acquisition of Plant Root Nitrogen-Fixing Symbiosis in Fabids Recruited the Same Genetic Pathway for Nodule Organogenesis

**DOI:** 10.1371/journal.pone.0064515

**Published:** 2013-05-31

**Authors:** Sergio Svistoonoff, Faiza Meriem Benabdoun, Mathish Nambiar-Veetil, Leandro Imanishi, Virginie Vaissayre, Stella Cesari, Nathalie Diagne, Valérie Hocher, Françoise de Billy, Jocelyne Bonneau, Luis Wall, Nadia Ykhlef, Charles Rosenberg, Didier Bogusz, Claudine Franche, Hassen Gherbi

**Affiliations:** 1 Equipe Rhizogenèse, UMR DIADE (IRD, UM2), Institut de Recherche pour le Développement, Montpellier, France; 2 Departement of Biology and Ecology, Mentouri University, Constantine, Algeria; 3 Plant Biotechnology Division, Institute of Forest Genetics and Tree Breeding, Coimbatore, India; 4 Laboratorio de Bioquímica, Microbología e Interacciones Biológicas en el Suelo L, Departamento de Ciencia y Tecnología, Universidad Nacional de Quilmes, Bernal, Argentina; 5 Biologie et Génétique des Interactions Plante-Parasite (INRA, CIRAD, SupAgro), Campus International de Baillarguet, Montpellier, France; 6 Laboratoire Commun de Microbiologie (IRD/ISRA/UCAD), Dakar, Sénégal; 7 Laboratoire des Interactions Plantes Microorganismes (UMR 2594/441, CNRS/INRA), Castanet-Tolosan, France; Friedrich-Alexander-University Erlangen-Nurenberg, Germany

## Abstract

Only species belonging to the Fabid clade, limited to four classes and ten families of Angiosperms, are able to form nitrogen-fixing root nodule symbioses (RNS) with soil bacteria. This concerns plants of the legume family (*Fabaceae*) and *Parasponia (Cannabaceae)* associated with the Gram-negative proteobacteria collectively called rhizobia and actinorhizal plants associated with the Gram-positive actinomycetes of the genus *Frankia*. Calcium and calmodulin-dependent protein kinase (*CCaMK*) is a key component of the common signaling pathway leading to both rhizobial and arbuscular mycorrhizal symbioses (AM) and plays a central role in cross-signaling between root nodule organogenesis and infection processes. Here, we show that *CCaMK* is also needed for successful actinorhiza formation and interaction with AM fungi in the actinorhizal tree *Casuarina glauca* and is also able to restore both nodulation and AM symbioses in a *Medicago truncatula ccamk* mutant. Besides, we expressed auto-active *CgCCaMK* lacking the auto-inhibitory/CaM domain in two actinorhizal species: *C. glauca (Casuarinaceae),* which develops an intracellular infection pathway, and *Discaria trinervis* (*Rhamnaceae*) which is characterized by an ancestral intercellular infection mechanism. In both species, we found induction of nodulation independent of *Frankia* similar to response to the activation of *CCaMK* in the rhizobia-legume symbiosis and conclude that the regulation of actinorhiza organogenesis is conserved regardless of the infection mode. It has been suggested that rhizobial and actinorhizal symbioses originated from a common ancestor with several independent evolutionary origins. Our findings are consistent with the recruitment of a similar genetic pathway governing rhizobial and *Frankia* nodule organogenesis.

## Introduction

While more than 80% of land plants can establish root endosymbioses with *Glomeromycota* fungi through the arbuscular mycorrhizal (AM) association, only a small group of plants limited to four orders (Fagales, Fabales, Rosales and Cucurbitales) are able to form nitrogen-fixing root nodule symbiosis (RNS) in association with soil bacteria. This group comprises (i) most of the ∼20.000 legume species and a few species of *Parasponia (Cannabaceae)* able to interact with Gram-negative proteobacteria, collectively called rhizobia; and (ii) actinorhizal plants, ∼280 species able to interact with Gram-positive filamentous actinobacteria *Frankia* [1], [2]. Fagales, Fabales, Rosales and Cucurbitales constitute a single clade ( = Fabids), however, phylogenetic analyses show that nodulation is not an ancestral character, but was probably acquired independently at least nine times in different lineages of Fabids, pointing to a genetic predisposition towards nodulation in this clade [3]–[5].

In recent years, huge progress has been made in understanding signaling pathways leading to rhizobial nodulation in two model legumes, *Medicago truncatula* and *Lotus japonicus*. RNS is the result of a coordinated exchange of signals between the plant and the bacteria leading to the activation of two synchronized processes: bacterial infection and nodule primordium initiation. Nod factors (NFs), lipo-chito-oligosaccharides synthesized by rhizobia, are the key molecular determinants that enable the specific recognition of bacterial symbionts by the plant [6]. Their perception is mediated by LysM receptors and elicits several host cellular responses, notably intracellular calcium oscillations and the activation of a signaling cascade involving a specific set of genes [7]. Components of this signaling cascade include a leucine-rich repeat receptor-like kinase (*SYMRK/DMI2*), a putative cation channel (*DMI1/Castor/Pollux*), a putative nuclear pore component (*NUP, NENA*), a calcium and calmodulin-dependant kinase (*CCaMK/DMI3*) and a protein with a short coiled-coil domain (*IPD3/Cyclops*) [7]. Interestingly, AM fungi are able to synthesize molecules similar to NFs [8] and several components of the NF signaling pathway (SYM) are essential for AM formation, suggesting that legume nodulation evolved by recycling at least part of the ancestral and widespread program used by most plants to interact with AM fungi [9]. Elements of the SYM signaling pathway were recruited for nodulation in at least three other nodulating lineages. *SYMRK* was shown to be essential for the interaction with *Frankia* in two actinorhizal species: *Casuarina glauca* (Fagales) and *Datisca glomerata* (Cucurbitales) [10], [11]. In *Parasponia andersonii* (Rosales) the interaction with rhizobia depends on *NFP* (a LysM receptor kinase) and *CCaMK* [12].

The extent to which nodulators use components of the SYM signaling pathway is nevertheless poorly known and the recent discovery that some *Aeschynomene* species are able to interact with *Bradyrhizobium* without the intervention of NFs [13] demonstrates that alternative signaling pathways enabling nodulation exist even within the legume family. Canonical *Nod* genes are also absent in several *Frankia* genomes [14] again suggesting that only part of the SYM signaling pathway is involved in actinorhizal nodulation. Presumed orthologs of SYM genes are present in the transcriptome of actinorhizal plants during nodulation and mycorrhization [15], [16] but as mentioned above, direct evidence supporting their role in nodulation is only available for *SYMRK*.

In the present study, we characterized the *C. glauca CCaMK* gene. In legumes, *CCaMK* is genetically positioned immediately after *SYMRK* in the SYM signaling pathway. It is likely a decoder of calcium oscillations and plays a crucial role in the activation of the nodulation process and in cross-signaling between nodule organogenic and rhizobial infection pathways [17]–[21]. Here, we show that *CCaMK* is necessary for successful nodulation and mycorrhization of *C. glauca*. We also demonstrate that constitutive activation of *CCaMK* is sufficient to trigger nodule organogenesis in the absence of *Frankia* not only in *C. glauca* but also in *Discaria trinervis* (Rosales), a species characterized by an ancestral intercellular infection mechanism, suggesting that every independent acquisition of nodulation recruited the whole pathway beyond *SYMRK*.

## Results

### *CgCCaMK* Encodes a Calcium and Calmodulin Dependent Kinase Related to Symbiotic *CCaMKs*

Screening of an expressed sequence-tag database of *C. glauca* nodules [15] enabled identification of three ESTs corresponding to *CCaMK* gene transcripts. By cloning its full-length cDNA and the corresponding genomic fragment, we found that *CgCCaMK* encodes a 520 aa putative protein and has similar gene and protein structures compared to legume *CCaMKs*. The deduced amino acid sequence showed a high degree of similarity with several legumes (*L. japonicus, M. truncatula, S. rostrata*). Notably, similarity reached 89% with *S. rostrata* in the kinase domain, 93% with the 3 legume species in the autoinhibition/calmodulin-binding domain and up to 100% with the 3 legume species also in the EF-hand motifs (Fig. S1). A phylogenetic analysis including *CgCCaMK* and 43 similar coding sequences revealed that *CgCCaMK* belongs to a cluster comprising *CCaMKs* from legumes, cereals and solanaceous species involved in AM and/or RNS (Fig. S2). Interestingly, *CCaMKs* involved in RNS belong to the same strongly supported group, which does not include *CgCCaMK*.

### *C. glauca CgCCaMK* is Involved in Mycorrhization and in the Early Stages of Actinorhizal Nodulation

**Figure 1:**
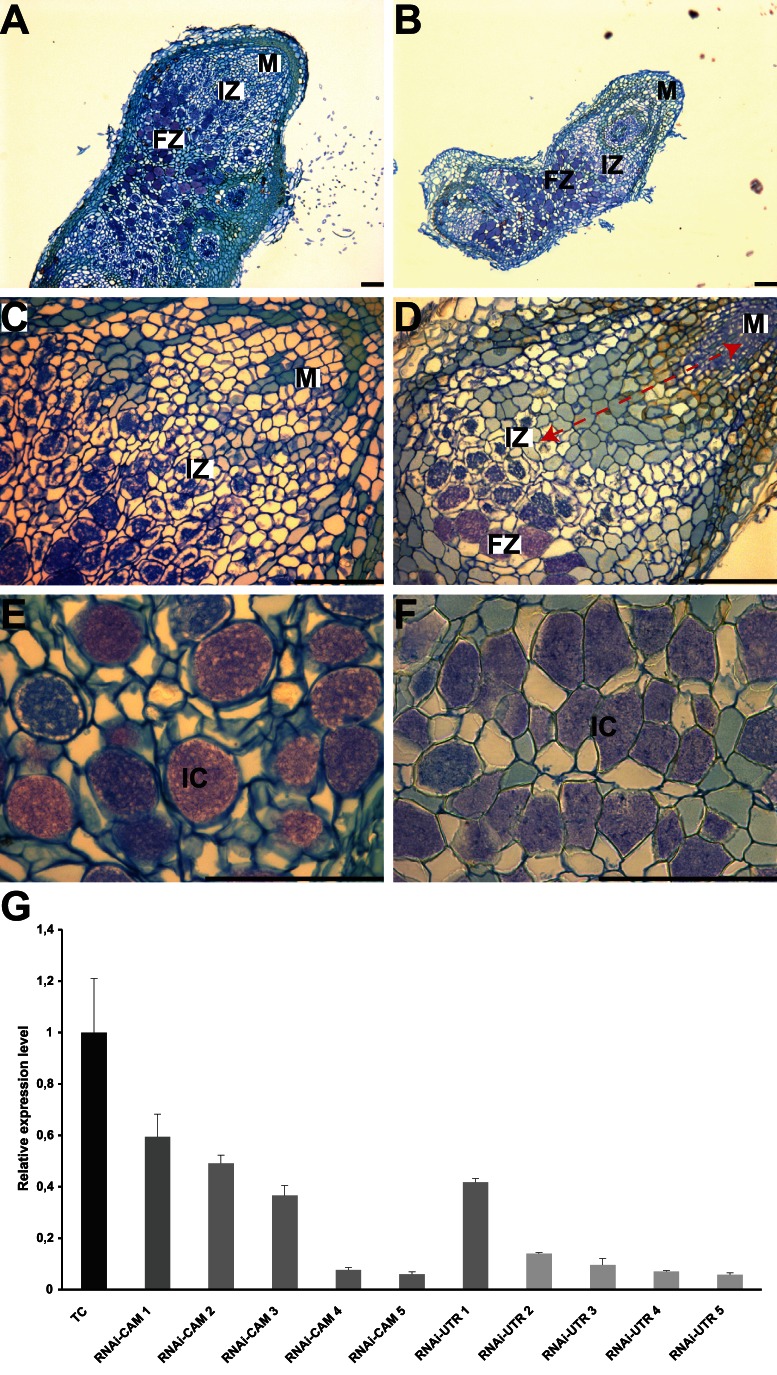
Knockdown phenotype of *CgCCaMK* after *Frankia* inoculation. (A-F) Longitudinal sections (6 µm) of nodule lobes stained with toluidine blue. (A, C and E) Transgenic control nodule. (B, D and F) *CgCCaMK*-RNAi 10 weeks after inoculation. Nodules in *CgCCaMK*-RNAi plants (B) were often smaller than in transgenic controls (A). The area between the meristem and the infection zone is wider in RNAi nodules (red arrow) (D). (E and F) Magnification of the fixation zone showing the presence of *Frankia* in cortical cells. FZ, fixation zone, IC, infected cells, IZ, infection zone, M, meristem. Bars = 100 µm. (G) Quantification of *CgCCaMK* mRNA levels in RNAi plants determined by real-time qPCR. Quantification was performed on 5 independent RNAi-CAM and 5 RNAi-UTR plants. *CgUbi* was used as a reference. The average of two independent non-transgenic control roots and three transgenic control roots is shown. Expression levels are relative to transgenic control roots. All error bars indicate standard errors of the mean of 3 technical replicates on different samples.

Using an RNAi approach, we investigated the symbiotic function(s) of *CgCCaMK*. We generated composite plants expressing RNAi constructs targeting either the 5′ UTR region (*RNAi-UTR*) or the calmodulin domain (*RNAi-CAM*) and compared their phenotype regarding nodulation and mycorrhization to control transgenic plants expressing the GFP selection marker only (TC). To analyze the effect on nodulation, 55 RNAi plants (24 *RNAi-UTR* and 31 *RNAi-CAM*) and 23 TC showing GFP expression were inoculated with *Frankia* and monitored for three months. Nodulation started from the third week in TC and from the fourth to the fifth week in RNAi plants. Ten weeks after inoculation, 60.87% of TC were nodulated, while only 41.94% and 37.50% of *RNAi-UTR* and *RNAi-CAM* plants respectively had nodules. RNAi plants formed fewer nodules than TC (Table 1) and the nodule phenotype ranged from small one-lobed to large multi-lobed nodules but no major alteration in the shape of the nodular lobe was observed. Quantitative RT-PCR was performed to test the efficiency of *CgCCaMK* knockdown in RNAi roots (10 RNAi plants) and revealed a reduction in endogenous *CgCCaMK* expression ranging from 58% to 94% for *RNAi-UTR* and from 40% to 94% for *RNAi-CAM*) (Fig. 1G). Moreover, the effects on nodulation were proportional to the reduction in endogenous *CgCCaMK* expression. Plants with low expression levels were unable to nodulate or formed only a few small nodules. To analyze the cytological organization of RNAi nodules, ten nodules were chosen from plants exhibiting small and few nodules. Semi-thin sections showed that RNAi nodules had the same features as TC (Fig. 1). However, the infection zone appeared to be larger and contained few infected cells (Fig. 1D) suggesting a role for *CgCCaMK* during the infection process. In addition, the hypertrophied infected cells of the fixation zone were smaller in the RNAi nodules than in TC (respectively 27% and 17% smaller in *RNAi-UTR* and *RNAi-CAM* than in TC; Fig. 1E and F). This suggests that *Frankia*-infected cells are not completely filled which is consistent with a role for *CgCCaMK* in the infection process. To analyze the effect of *CgCCaMK* knockdown on mycorrhization, composite plants showing strong GFP fluorescence in roots (15 *RNAi-UTR*, 20 *RNAi-CAM* and 10 TC) were inoculated with *Rhizophagus irregularis* and harvested nine weeks later. Fungal colonization of RNAi roots was very weak compared to that of TC (Table 2) and intraradical hyphae, vesicles and arbuscules were found at very low frequencies (from 0% to 10%) but no morphological differences between control and RNAi plants were detected. In conclusion, both nodulation and mycorrhization were more affected in RNAi plants than in TC plants and stronger differences were recorded in *RNAi-UTR* plants.

**Table 1:** Actinorhizal nodulation in transgenic control (23 *TC*) and *CgCCaMK* knock down roots (31 *RNAi-CAM* and 24 *RNAi-UTR*).

	Nodulated/total plants and mean number of nodules			>7 nodules/nodulated plants
Genotype	6 WPI	10 WPI	12 WPI	12 WPI
***TC***	21.75%	60.87%	65.22%	73.33%
	1.30 (±0.64)	6.87 (±1.94)	9.65 (±2.37)	
***RNAi-CAM***	16.13%	41.94%	54.84	47.06%
	0.30 (±0.75)	4.06 (±1.42)	6.10 (±1.70)	
***RNAi-UTR***	12.5%	37.50	58.33%	28.57%
	0.29 (±0.18)	2.21 (±0.90)	3.83 (±1.27)	

The percentage of nodulation and the average number of nodules was calculated at 6, 10 and 12 weeks post inoculation (WPI). Standard errors are in brackets.

**Table 2:** *Rhizophagus irregularis* ( = *Glomus intraradices*) mycorrhization of transgenic control and *CgCCamK* knock-down roots.

	Number of plants	Mycorrhization frequency (%)
***TC***	10	41.01 (±8.49)
***RNAi-CAM***	20	8.09 (±2.14)
***RNAi-UTR***	15	5.03 (±1.39)

Mycorrhization frequency corresponds to the percentage of mycorrhized roots in 10 TC (transgenic control) plants, 20 RNAi-CAM plants and 15 RNAi-UTR plants evaluated using the gridline intercept method. Mycorrhization frequency was calculated using roots harvested 9 weeks post inoculation. Standard errors are in brackets.

### *CgCCaMK* is Able to Fully Complement the *M. truncatula dmi3* Mutant

**Figure 2:**
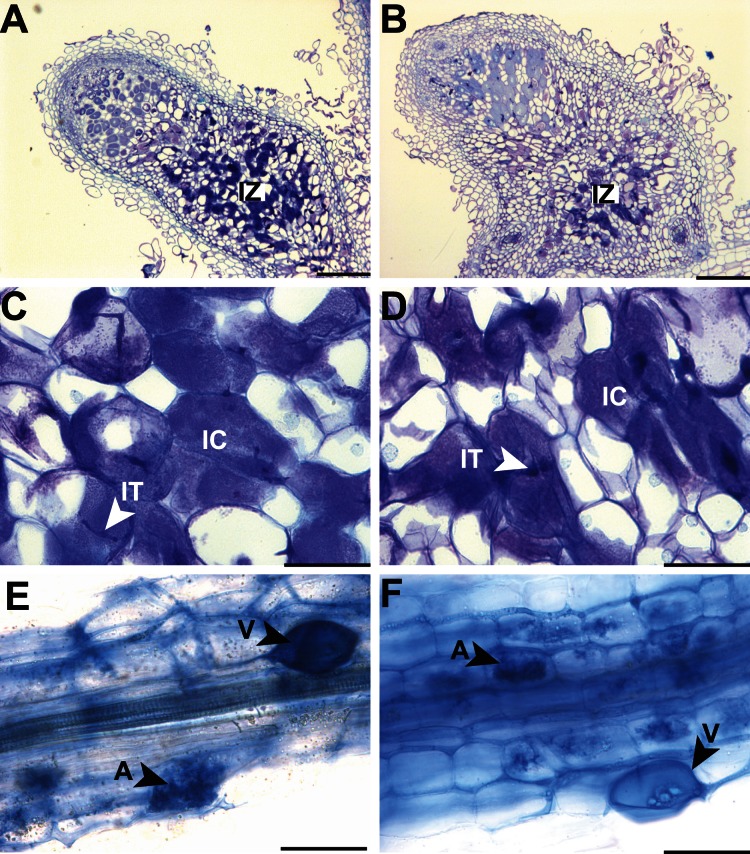
Complementation of *M. truncatula dmi3* (TRV25) mutant with the *CgCCaMK* coding sequence under the control of the *MtDMI3* promoter. The *mtdmi3* mutant was transformed with the control *ProMtDMI3::MtDMI3* construct (A, C and E) or the *ProMtDMI3::CgCCaMK* construct (B, D and F). (A, B, C and D) semi-thin sections (6 µm) of nodules stained with toluidine blue. Nodules were obtained after inoculation with *Sinorhizobium meliloti*. Infected cells and infection threads are visible (arrows). (E and F) Cleared roots 6 weeks after inoculation with *Rhizophagus irregularis* stained with trypan blue. Fungal hyphae grew through the epidermis and exodermis and formed arbuscules and vesicles in the inner root cortex. IC: infected cells; IT: infection thread; IZ: infection zone; A: arbuscule; V: vesicle. Bars = 500 µm (A and B); 50 µm (C, D, E and F).

To determine whether *CgCCaMK* is functionally equivalent to its homolog in *M. truncatula*, we introduced a *CgCCaMK* cassette (P*MtDMI3::CgCCaMKCDS::*T*MtDMI3*) in the *M. truncatula dmi3* mutant [22], [23]. Composite plants were then inoculated either with *S. meliloti* or *R. irregularis* to test their symbiotic performance. *mtdmi3* plant mutants expressing the *CgCCaMK* cassette showed a wild type nodulation phenotype (five weeks after inoculation, nodules were present in 9 out of 10 tubes, with an average of 14,4 nodules/tube) comparable to the positive control (nodules in 7 out of 9 tubes, with an average of 11,6 nodules/tube). Nodules exhibited a typical structure with a central zone containing infected cells including the presence of infection threads (Fig. 2A and B and C and D). Also, no significant differences in acetylene reduction activity (ARA) were detected between complemented *dmi3* mutants (39.75±13.85 Units) and positive controls (33.50±18.10 Units), while only very low amounts of ethylene (1.50±0.29 Units) were detected in negative control plants. Furthermore, *dmi3* mutants carrying the *CgCCaMK* cassette were efficiently colonized by AM fungi. Mycorrhizal structures including arbuscules, hyphae, vesicles and spores were observed and were very similar to those detected in positive controls (Fig. 2E and F). Taken together, our results demonstrate that *CgCCaMK* restores nodulation, nitrogen fixation and AM in *mtdmi3* mutant.

### Truncated *CgCCaMK* Initiates Nodule Organogenesis in the Absence of *Frankia* in *C. glauca*

**Figure 3:**
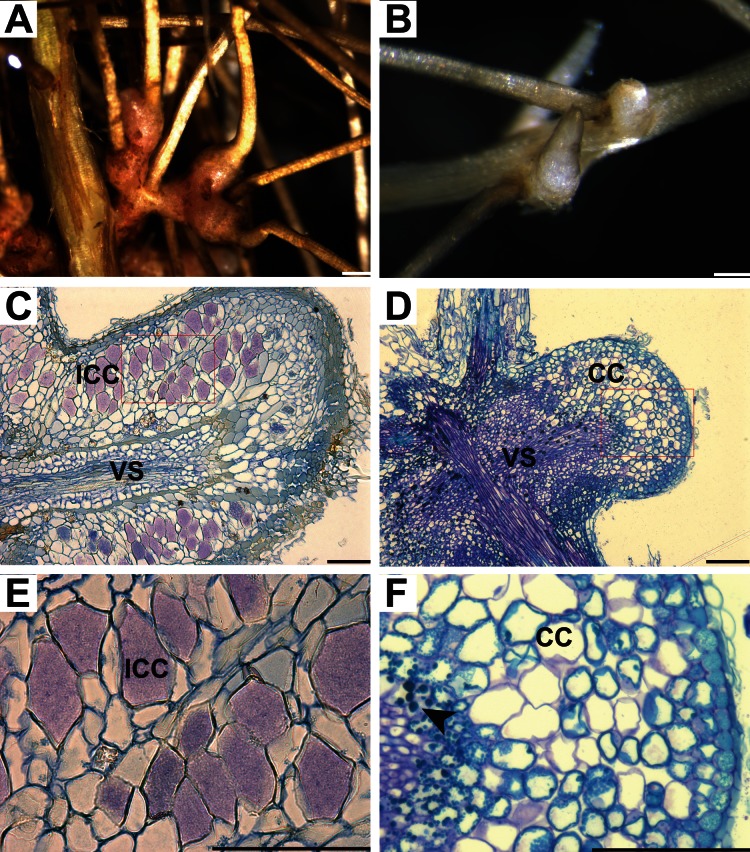
Induction of spontaneous nodules in *C. glauca* roots expressing truncated *CgCCaMK* constructs. (A, C and E) *Frankia*-infected nodule. (B, D and F) Spontaneous nodule. (A) Multi-lobed nodule 4 weeks after *Frankia* inoculation. (B) Small one-lobed nodule on hairy roots formed two months after transfer to hydroponics. (C, D, E and F) semi-thin sections (6 µm) of nodules stained with toluidine blue. (C) Section of a nodule lobe showing infected cortical cells and vascular system. (E) A close up of area in (C) showing hypertrophied cells containing *Frankia*. (D) Section of a spontaneous nodule harboring a poorly developed central vascular system and numerous cortical cell layers. (F) Close up of area in (D) showing that cortical cells are not hypertrophied and are free of bacteria. Phenolic compounds tend to accumulate in the form of droplets (arrow). CC, cortical cells; ICC, infected cortical cells; VS, vascular system. Bars = 500 µm (A and B); 100 µm (C, D, E and F).

Spontaneous nodule formation in the absence of symbiotic bacteria has been obtained in several plants able to form nodules in interaction with rhizobia by expressing dominant active forms of legume *CCaMKs* including the truncated versions lacking the auto-inhibitory/CaM domain [24], [25]. To investigate whether an auto-active form of *CgCCaMK* is also able to elicit organogenesis of actinorhizal nodules, we generated three constructs driven by the *CgCCaMK* promoter (1) Δ*307* corresponding to the kinase domain of *CgCCaMK*, (2) Δ*322* corresponding to the kinase domain and the region before autoinhibition/CaM domain of *CgCCaMK* and (3) a control cassette corresponding to the whole *CgCCaMK* coding sequence (Fig. S3). These constructs were first introduced in *C. glauca*. For a period of six months, composite plants were grown hydroponically at low nitrogen concentrations and roots were examined every week. Nodule-like structures began to appear during the second month, mainly on the lower half of the root system in 20% (n = 4/20) and 26% (n = 7/27) of plants expressing Δ*307* and Δ*322* respectively but were never observed in control plants. The number of nodules ranged from 1 to 9 per plant. These spontaneous nodules were generally small and round-shaped, uni-lobed and did not show the characteristic nodular roots that are usually formed at the apex of *C. glauca* nodules (Fig. 3A and D). In contrast to typical *C. glauca* nodules, these structures were still small six months after their appearance. Semi-thin sections performed on nodule-like structures showed the presence of a central vascular system and several layers of cortical cells, two typical features of actinorhizal nodules (Fig. 3D). However cortical cells were not hypertrophied and did not contain any visible *Frankia* filaments (Fig. 3E and F). Compared to *C. glauca* nodules containing *Frankia* (Fig. 3C), spontaneous nodules were also smaller, not elongated, their vascular system was less developed (Fig. 3D) and contained higher amounts of phenolic compounds in the form of droplets mainly in the endodermis and cortex (Fig. 3F). These results indicate that the constitutive kinase activity of truncated *CgCCaMK* is sufficient to induce the first steps of nodule organogenesis in the absence of *Frankia* but an additional signal is probably needed to fully develop and maintain a mature nodule.

### Truncated *CgCCaMK* Initiates and Maintains Nodule Organogenesis in the Absence of *Frankia* in *D. trinervis*

**Figure 4:**
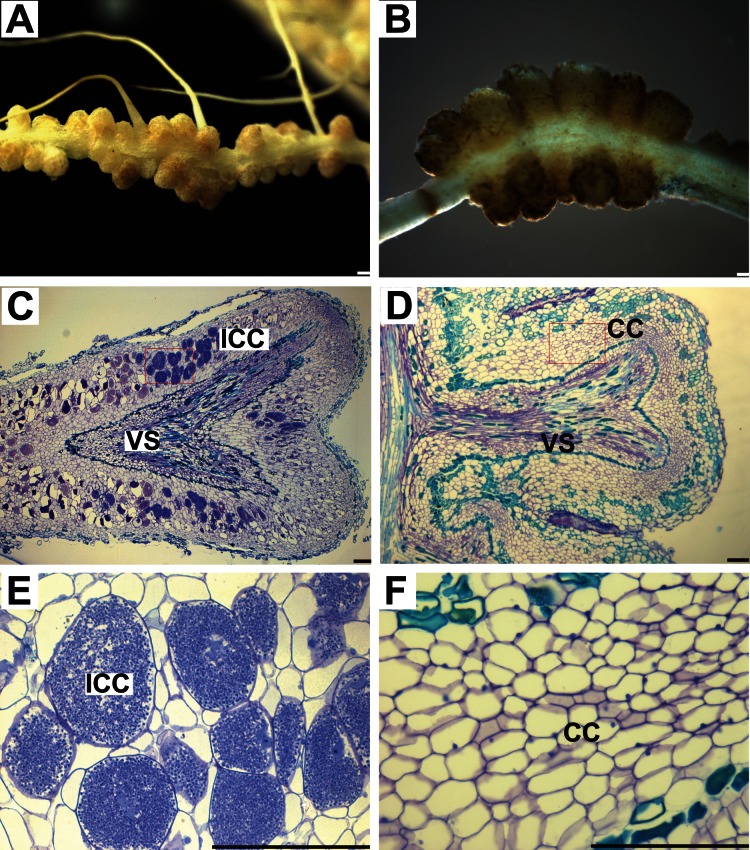
Induction of spontaneous nodules in *D. trinervis* roots expressing truncated *CgCCaMK* constructs. (A) Multi-lobed nodules 4 weeks after inoculation with *Frankia*. (B) Multi-lobed spontaneous nodules on hairy roots formed two months after transfer to hydroponics. (C, D, E and F) semi-thin sections (6 µm) of nodules stained with toluidine blue. (C and E) Section of wild-type nodule harboring two lobes, a central vascular system (VS) and numerous infected cortical cells (ICC). (E) A close up of area in (C) showing the hypertrophied cells containing *Frankia*. (D and F) Section of a spontaneous nodule. Two lobes and a central vascular system are visible. (F) A close up of area in (D). Cortical cells are not hypertrophied and do not contain *Frankia*. CC, cortical cells; ICC, infected cortical cells; VS, vascular system. Bars = 500 µm (A and B); 100 µm (C, D, E and F).

*D. trinervis* is an actinorhizal Rosales infected by *Frankia* through the primitive intercellular infection pathway without the formation of infection threads [26]. To examine whether this phylogenetically distant and primitive symbiosis also recruits the signaling pathway mediated by *CgCCaMK,* we generated transgenic *D. trinervis* plants expressing the *CgCCaMK* constructs described above and grew them hydroponically with a low nitrogen concentration. Like for *C. glauca*, nodule-like structures began to appear during the second month only in plants expressing truncated *CgCCaMKs* (n = 6/31). Strikingly, spontaneous *D. trinervis* nodules were generally numerous, multilobed, distributed on a normal looking root system, had an indeterminate growth and displayed the same shape and size as typical *Frankia*-induced nodules (Fig. 4A to D). Examination of semi-thin sections confirmed these similarities: spontaneous nodules showed the usual features of a *Frankia*-infected nodule except that cortical cells were not hypertrophied and did not contain *Frankia* (Fig. 4E and F). In contrast to *C. glauca*, accumulation of phenolic compounds was limited to the vasculature and external cortical cell layers, the apical meristem appeared to be functional and nodules were often multi-lobed, a typical anatomic characteristic of mature *D. trinervis* nodules (Fig. 4F). These observations indicate that the activation of *CgCCaMK* is sufficient to initiate and maintain indeterminate nodule organogenesis in *D. trinervis*.

## Discussion

In legumes, *CCaMK* plays a central role in the cross-talk between nodule organogenesis and bacterial infection [17]–[19]. *CCaMK* null mutants are unable to develop RNS and AM [23], [25], [27]. In contrast, dominant active *CCaMKs*, which have constitutive phosphorylation activity, trigger nodulation in the absence of bacteria or bacterial signals [24], [25].

We isolated and characterized the *CCaMK* gene from the actinorhizal tree *C. glauca*. Sequence analysis revealed that *CgCCaMK* has all the features of other plant *CCaMKs*. We found that *CgCCaMK* is able to fully complement the *M. truncatula dmi3* mutant for mycorrhization and that downregulation of *CgCCaMK* strongly affects AM interaction, confirming the results of previous studies on legumes and rice ([28]. Recently, the mycorrhization phenotype of *dmi3* mutants was fully complemented with *CCaMK* genes from hornworts and liverworts, two lineages that diverged from legumes more than 400 MY ago, suggesting that a strong selective pressure probably related to the widespread occurrence of AM associations among land plants maintained the key ancestral features of *CCaMK* [29].

Regarding the formation of actinorhizal nodules, we show here that downregulation of *CgCCaMK* leads to a delay in nodulation, a decrease in the number of nodules, a reduction in their size, and interferes with the infection process. Downregulation of *CCaMKs* leads to similar results in legumes [30] pointing to an universal role for *CCaMKs* in rhizobial and actinorhizal root nodule symbioses. We further confirmed that *CgCCaMK* is functionally equivalent to *MtCCaMK* by achieving full complementation of the *dmi3* null mutant for rhizobial infection, nodule organogenesis, and nitrogen fixation. *CCaMK* genes from non-legumes including the above mentioned liverworts, hornworts, lily, and rice have been shown to complement the nodulation phenotype of *dmi3*. However, complementation was always partial, suggesting that additional tweaks are needed to fully restore nodulation in *M. truncatula*. Several amino acids in auto-inhibitory/CaM domains and/or in EF-hand motifs of CCaMK are conserved among nodulating species (S322, W338, S340, F342, K348, S351, T446; Fig. S1) and may be essential to achieve a full complementation. Previous work performed on *SYMRK* indicated that full complementation is only achieved with genes from nodulating plants, with the exception of *SYMRK* from *Tropaeolum* [11]. It would be interesting to know if *CCaMKs* from asterids (*Petunia*, tomato) are able to fully complement *dmi3*. In contrast to *M. truncatula*, the *L. japonicus* mutant *ccamk-3* (a point mutation that causes a glycine to glutamic acid substitution in the kinase domain at position 30) [25] can be fully complemented with a rice *CCaMK* gene [31]. Less stringent requirements in *L. japonicus* may be related to the presence in this species of the primitive intercellular infection pathway [18] which has not been described in *M. truncatula*.

To further investigate the role of *CCaMK* in the activation of nodulation in actinorhizal species, we used two deregulated forms of *CgCCaMKs* lacking the auto-inhibitory/CaM domain ( = Δ*307* and Δ*322)*. In *C. glauca*, *CgCCaMKs,* Δ*307* and Δ*322* were able to trigger nodule organogenesis in the absence of *Frankia,* demonstrating that *CCaMK* activation is sufficient to induce all the pathways required for nodule initiation. Spontaneous nodules were mostly small, not branched and had a limited vascular system. Nodule initiation in actinorhizal species infected through intracellular infection mode, including *C. glauca* always shows the formation of the prenodule. The prenodule is the result of limited cell divisions triggered in the cortex near the infection site but is not directly involved in nodule formation and its actual function is unknown [32]. It is possible that bacterial infection is needed to fulfill nodule development. Furthermore, we addressed the question of whether the SYM pathway is involved in nodule organogenesis in a species colonized through intercellular infection. To this end, we introduced the dominant active *CgCCaMK* Δ*307* and Δ*322* in *D. trinervis*, an actinorhizal plant phylogenetically distant from *C. glauca,* infected through the primitive intercellular infection pathway without the involvement of infection threads. Surprisingly, we observed the formation of spontaneous nodules phenotypically similar to *Frankia*-infected nodules except that they were free of bacteria. In *D. trinervis*, nodule formation does not include the prenodule step and nodules develop until maturity before intracellular infection occurs [26]. Spontaneous nodule formation was recently obtained by introducing an auto-active *CCaMK* from *M. truncatula* in *P. andersonii*, the only non-legume able to form a RNS with rhizobia [12]. Moreover, it has been recently proposed that rhizobial infection through direct intercellular epidermal invasion constitutes the ground state of bacterial invasion from which crack entry and RH invasion modes might have subsequently evolved [18].

The ability to establish RNS was most probably acquired independently by the ancestors of *P. andersonii*, *M. truncatula*, *C. glauca* and *D. trinervis* [5]. Our results suggest that the activation of *CCaMKs* is a central feature allowing nodulation and was recruited independently at least 4 times during the evolution of RNS. As a result *CCaMKs* are now central players of RNS regardless of the symbiotic partners, the infection mechanisms or the anatomy of the symbiotic nodules.

## Materials and Methods

### Biological Materials

*C. glauca* seeds were purchased from Carter Seeds (California, USA) and grown as described in [33]. *D. trinervis* seeds were collected at Pampa de Huenuleo (Bariloche, Argentina) and were grown as described in [34]. Seeds of *M. truncatula* cv Jemalong A17 wild-type and *TRV25 dmi3* mutant were grown according to [23]. *A. rhizogenes* strain A4RS [35] was used for hairy root transformation of *C. glauca* and *A. rhizogenes* strain ARqua1 [36] was used for both *M. truncatula* and *D. trinervis* [34]. *Frankia* strains CcI3 [14] and BCU110501 [37] were used to nodulate *C. glauca* [38] and *D. trinervis* [34] respectively. *Sinorhizobium meliloti* strain RCR2011 was used to nodulate *M. truncatula*. *Rhizophagus irregularis ( = Glomus intraradices* DAOM 197198) starting cultures were kindly provided by G. Bécard (Cell Surfaces and Plant Signaling, UMR CNRS - Paul Sabatier University, Toulouse, France). The fungal inoculum was used for both *C. glauca* and *M. truncatula* mycorrhization.

### Isolation of *CgCCaMK*

A full length *CgCCaMK* fragment was amplified from a nodule cDNA using *CgCCaMK-For1*: AGAGTGCTGGCGAAGCCATGCATG and *CgCCaMK-Rev4*: GTATGCCATGAAGGAAAACAGCTCC primers designed from *C. glauca* nodule EST library [15] homologous to the *CCaMK* gene. The *CgCCaMK* genomic fragment was amplified on total DNA from a *C. glauca* young shoot apex using the same primer pair. Amplified fragments were then cloned into pGEM-T vector (Promega) and sequenced. The *CgCCaMK* promoter (2165 bp) was cloned using the Universal Genome Walker kit (Clontech) applied to genomic DNA as recommended by the manufacturer.

### Plant Transformation Procedures

*A. rhizogenes*-mediated transformation of *C. glauca*, *D. trinervis* and *M. truncatula* was performed as described in [39], [34], [36] respectively. Selection of transgenic roots was based on GFP fluorescence. For nodulation studies, plants were grown as described in [40], [41], [36]. For spontaneous nodule induction, *C. glauca* and *D. trinervis* hairy root plants were grown in BD medium [40] with 0.5 mM of NH_4_NO_3_. For mycorrhization analyses, plants were grown as described in [41].

### RNAi, Complementation and Truncated *CCaMKs*

To generate the RNAi constructs, *RNAi-UTR* and *RNAi-CAM* fragments corresponding to respectively 350 bp of the *CgCCaMK* untranslated region and to 356 bp of the calmodulin binding domain were amplified using *Att*B1-*CgCCaMKRNAiUTR*-F3/*Att*B2-*CgCCaMKRNAiUTR*-R4 primers and *Att*B1-*CgCCaMKRNAiCAM*-F1/*Att*B2-*CgCCaMKRNAiCAM*-R2 primers (see SI). RNAi fragments were then cloned into the pHKN29 binary vector [42] modified by introducing the Gateway (Invitrogen) RNAi cassette from the pHellsgate12 vector [43] at *Spe*I/*Sac*I sites. For functional complementation, the *CgCCaMK* coding sequence (1563 bp) was fused to the *MtDMI3* promoter (1048 bp) and terminator (320 bp) and cloned into the pCambia 2202 binary vector. The pCambia2202 containing the whole *MtDMI3* genomic fragment including the promoter (1048 bp) and terminator (320 bp) was used as positive control. To generate truncated *CgCCaMK*, constructs were prepared by multiple cloning steps in pGEM-T easy vector (Promega). The *CgCCaMKs* Δ*307,* Δ*322* and *CDS* fragments were fused to the *CgCCaMK* promoter and to the *NOS* terminator. The cassettes were then cloned in pHKN29.

### Expression Analysis of *CgCCaMK* Transcripts

Total RNA was extracted from roots using the RNEasy Plant MiniKit (Qiagen) and quantified using a NanoDrop ND-1000 spectrophotometer. One hundred ng per sample were reverse transcripted using SuperScriptIII H^-^ reverse transcriptase (Invitrogen). Real time qPCR was then performed as described in [10] with the following primers *qCgCCaMK-F*: ATGTCGTCGTTGGTTCCTC and *qCgCCaMK*-*R*: CTTCTTCCTTGCTGATGTATCC.

### Histochemical Analyses, Microscopy and Acetylene Reduction Assay (ARA)

*C. glauca* roots and nodules were fixed and dehydrated as described in [41]. *M. truncatula* nodules were fixed in a solution containing 50% EtOH, 4% formaldehyde and 5% acetic acid and dehydrated in 70% EtOH. Samples were embedded in Technovit 7100 resin (Heraeus Kulzer) as recommended by the manufacturer. Thin sections (6-µm) were cut with a HM355S microtome (Microm). Sections were colored with toluidine blue and mounted in Clearium Mountant (Surgipath). Samples were observed under a DMRB microscope (Leica) or a stereomicroscope MZFLIII (Leica) equipped with a MP5 (Qimaging) digital camera. Cell surface areas of the fixation zone (from 10 different RNAi nodules and 5 TC nodules, 10 to 20 hypertrophied infected cells/nodule) were measured using Region-Of-Interest (ROI) in ImageJ software (http://rsb.info.nih.gov/ij/). To visualize and quantify AM structures in *C. glauca* and *M. truncatula*, roots were prepared and observed as described in [10], [44]. To assess nitrogenase activity, acetylene reduction assays (ARA) of *M. truncatula* composite plants (8 plants per construct) were tested according to [45]. The amount of ethylene produced in each tube was assessed by measuring the height of the peak on the chromatogram, expressed in arbitrary units.

### Phylogeny Analysis

Sequences similar to CgCAMK were retrieved from GenBank or Phytozome (www.phytozome.org). Coding sequences were aligned using MAFFT [46]. The alignment was curated using trimAL [47]. A phylogenetic tree was calculated by maximum likelihood using PhyML 3.0 [48] with the following options: BIONJ starting tree, best searching method, estimated parameters for gamma distribution, proportion of invariable sites and transition/transversion ratio, a GTR substitution model and six gamma distributed discrete rates of evolution. One thousand non-parametric bootstrap replications were used to evaluate statistical support for branches. Rooting was performed using the midpoint method implemented in FigTree (http://tree.bio.ed.ac.uk/software/figtree/).

## Supporting Information

Figure S1 Amino acid sequence alignment of CgCCaMK, *M. truncatula* DMI3, *L. japonicus* CCaMK, *S. rostrata* CCaMK and *O. sativa* CCaMK. Identical and similar residues are shaded. The different protein domains are indicated by boxes: kinase domain (gray), calmodulin binding domain (black), EF hands (empty boxes). The autophosphorylation site is indicated by an asterisk. The alignment was performed using MAFFT software [46] and edited with CLC Sequence Viewer (http://www.clcbio.com//) software.
(PDF)

Figure S2 Maximum likelihood phylogeny of Calcium-Dependent Protein Kinases (*CDPKs*) similar to *CgCCaMK*. Branches with less than 50% bootstrap support were collapsed. All plant *CCaMKs* cluster together; other distantly related CDPK from Rosids and *Selaginella* were also included as an outgroup. Corresponding accession numbers are listed in Supporting Information section.
(PDF)

Figure S3 Schematic representation of truncated *CgCCaMK* constructs. Gray box: kinase domain; black box: calmodulin binding domain; empty box: EF hand.
(PDF)

File S1 Primer sequences. Accession numbers.
(DOC)
