# Supplementary material for: The Independent Acquisition of Plant Root Nitrogen-Fixing Symbiosis in Fabids Recruited the Same Genetic Pathway for Nodule Organogenesis
Source: PLoS One. 2013 May 31;8(5):e64515. doi: 10.1371/journal.pone.0064515 (PMC3669324; doi:10.1371/journal.pone.0064515)
Supplement: Figure S1 — Amino acid sequence alignment of CgCCaMK, M. truncatula DMI3, L. japonicus CCaMK, S. rostrata CCaMK and O. sativa CCaMK. Identical and similar residues are shaded. The different protein domains are indicated by boxes: kinase domain (gray), calmodulin binding domain (black), EF hands (empty boxes). The autophosphorylation site is indicated by an asterisk. The alignment was performed using MAFFT software [46] and edited with CLC Sequence Viewer (http://www.clcbio.com//) software. (PDF) [file pone.0064515.s001.pdf]

Figure S1

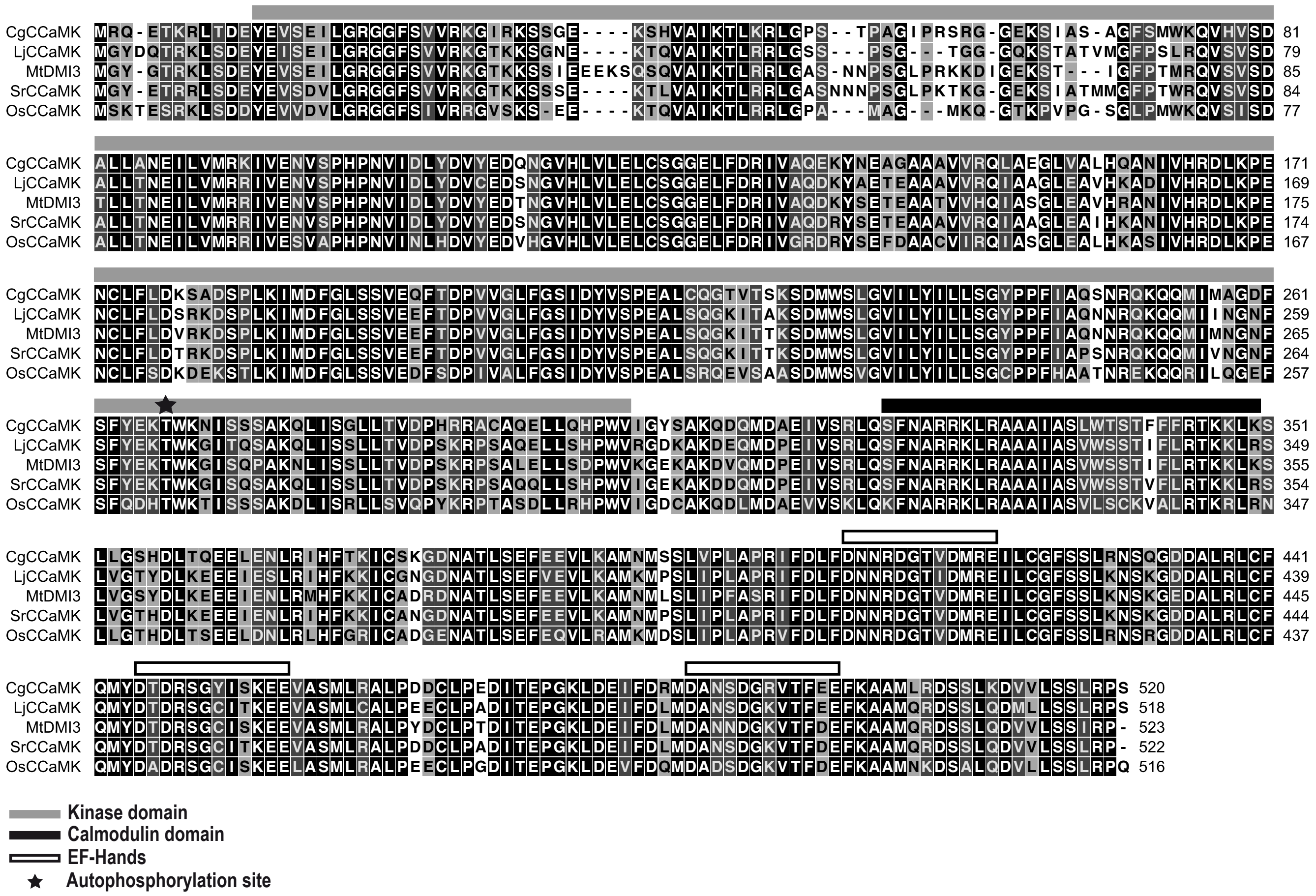
CgCCaMK MRQ-ETKRLTDEYEVSEILGRGGFSVVRKGIRKSSGE----KSHVAIKTLKRLGPS--TPAGIPRSRG-GEKSIAS-AGFSMWKQVHVSD 81
LjCCaMK MGYDQTRKLSDEYEISEILGRGGFSVVRKGTKKSGNE----KTQVAIKTLRRLGSS---PSG--TGG-GQKSTATVMGFPSLRQVSVSD 79
MtDMI3 MGY-GTRKLSDEYEVSEILGRGGFSVVRKGTKKSSIEEEKSQSQVAIKTLRRLGAS-NNPSGLPRKKDIGEKST---IGFPTMRQVSVSD 85
SrCCaMK MGY-ETRRLSDEYEVSDVLGRGGFSVVRKGTKKSSSE----KTLVAIKTLRRLGASNNNPSGLPKTKG-GEKSIATMMGFPTWRQVSVSD 84
OsCCaMK MSKTESRKLSDDYEVVDVLGRGGFSIVRRGVSKS-EE----KTQVAIKTLRRLGPA---MAG--MKQ-GTKPVPG-SGLPMWKQVSISD 77
CgCCaMK ALLANEILVMRKIVENVSPHPNVIDLYDVYEDQNGVHLVLELCSGGELFDRIVAQEKYNEAGAAAVVRQLAEGLVALHQANIVHRDLKPE 171
LjCCaMK ALLTNEILVMRRIVENVSPHPNVIDLYDVCEDSNGVHLVLELCSGGELFDRIVAQDKYAETEAAAVVRQIAAGLEAVHKADIVHRDLKPE 169
MtDMI3 TLLTNEILVMRRIVENVSPHPNVIDLYDVYEDTNGVHLVLELCSGGELFDRIVAQDKYSETEAATVVHQIASGLEAVHRANIVHRDLKPE 175
SrCCaMK ALLTNEILVMRRIVENVSPHPNVIDLYDVYEDSNGVHLVLELCSGGELFDRIVAQDRYSETEAAAVVRQIAAGLEAIHKANIVHRDLKPE 174
OsCCaMK ALLTNEILVMRRIVESVAPHPNVINLHDVYEDVHGVHLVLELCSGGELFDRIVGRDRYSEFDAACVIRQIASGLEALHKASIVHRDLKPE 167
CgCCaMK NCLFLDKSADSPLKIMDFGLSSVEQFTDPVVGLFGSIDYVSPEALCQGTVTSKSDMWSLGVILYILLSGYPPFIAQSNRQKQQMIMAGDF 261
LjCCaMK NCLFLDSRKDSPLKIMDFGLSSVEEFTDPVVGLFGSIDYVSPEALSQGKITAKSDMWSLGVILYILLSGYPPFIAQNNRQKQQMIINGNF 259
MtDMI3 NCLFLDVRKDSPLKIMDFGLSSVEEFTDPVVGLFGSIDYVSPEALSQGKITTKSDMWSLGVILYILLSGYPPFIAQNNRQKQQMIMNGNF 265
SrCCaMK NCLFLDTRKDSPLKIMDFGLSSVEEFTDPVVGLFGSIDYVSPEALSQGKITTKSDMWSLGVILYILLSGYPPFIAPSNRQKQQMIVNGNF 264
OsCCaMK NCLFSDKDEKSTLKIMDFGLSSVEDFSDPIVALFGSIDYVSPEALSRQEVSAASDMWSVGVILYILLSGCPPFHAATNREKQQRILQGEF 257
CgCCaMK SFYEKTWKNISSSAKQLISGLLTVDPHRRACAQELLQHPWVIGYSAKQDQMDAEIVSRLQSFNARRKLRAAAIASLWTSTFFFRTKKLKS 351
LjCCaMK SFYEKTWKGITQSAKQLISSLLTVDPSKRPSAQELLSHPWVRGDKAKDEQMDPEIVSRLQSFNARRKLRAAAIASVWSSTIFLRTKKLRS 349
MtDMI3 SFYEKTWKGISQPAKNLISSLLTVDPSKRPSALELLSDPWVKGEKAKDVQMDPEIVSRLQSFNARRKLRAAAIASVWSSTIFLRTKKLKS 355
SrCCaMK SFYEKTWKGISQSAKQLISSLLTVDPSKRPSAQQLLSHPWVIGEKAKDDQMDPEIVSRLQSFNARRKLRAAAIASVWSSTVFLRTKKLRS 354
OsCCaMK SFQDHTWKTISSSAKDLISRLLSVQPYKRPTASDLLRHPWVIGDCAKQDLMDAEVVSKLQKFNARRKLRAAAIASVLSCKVALRTKRLRN 347
CgCCaMK LLGSHDLTQEELENLRIHFTKICSKGDNATLSEFEEVLKAMNMSSLVPLAPRIFDLFDNNRDGTVDMREILCGFSSLRNSQGDDALRLCF 441
LjCCaMK LVGTYDLKEEEIESLRIHFKKICGNGDNATLSEFVEVLKAMKMPSLIPLAPRIFDLFDNNRDGTIDMREILCGFSSLKNSKGDDALRLCF 439
MtDMI3 LVGSYDLKEEEIENLRMHFKKICADRDNATLSEFEEVLKAMNMLSLIPFASRIFDLFDNNRDGTVDMREILCGFSSLKNSKGEDALRLCF 445
SrCCaMK LVGTHDLKEEEIENLRIHFKKICANGDNATLSEFEEVLKAMNMPSLIPLAPRIFDLFDNNRDGTVDMREILCGFSSLKNSKGDDALRLCF 444
OsCCaMK LLGTHDLTSEELDNLRLHFGRICADGENATLSEFEQVLRAMKMDSLIPLAPRVFDLFDNNRDGTVDMREILCGFSSLRNSRGDDALRLCF 437
CgCCaMK QMYDTDRSGYISKEEVASMLRALPDDCLPEDITEPGKLDEIFDRMDANSDGRVTFEEFKAAMLRDSSLKDVVLSSLRPS 520
LjCCaMK QMYDTDRSGCITKEEVASMLCALPEECLPADITEPGKLDEIFDLMDANSDGKVTFEEFKAAMQRDSSLQDMLLSSLRPS 518
MtDMI3 QMYDTDRSGCISKEEVASMLRALPYDCLPTDITEPGKLDEIFDLMDANNDGKVTFDEFKAAMQRDSSLQDVVLSSIRP- 523
SrCCaMK QMYDTDRSGCITKEEVASMLRALPDDCLPADITEPGKLDEIFDLMDANSDGKVTFDEFKAAMQRDSSLQDVVLSSLRP- 522
OsCCaMK QMYDADRSGCISKEELASMLRALPEECLPGDITEPGKLDEVFDQMDADSDGKVTFDEFKAAMNKDSALQDVLLSSLRPQ 516
Kinase domain
Calmodulin domain
EF-Hands
Autophosphorylation site
