# Supplementary figures and images for: The Independent Acquisition of Plant Root Nitrogen-Fixing Symbiosis in Fabids Recruited the Same Genetic Pathway for Nodule Organogenesis
Source: PLoS One. 2013 May 31;8(5):e64515. doi: 10.1371/journal.pone.0064515 (PMC3669324; doi:10.1371/journal.pone.0064515)

Figure S2

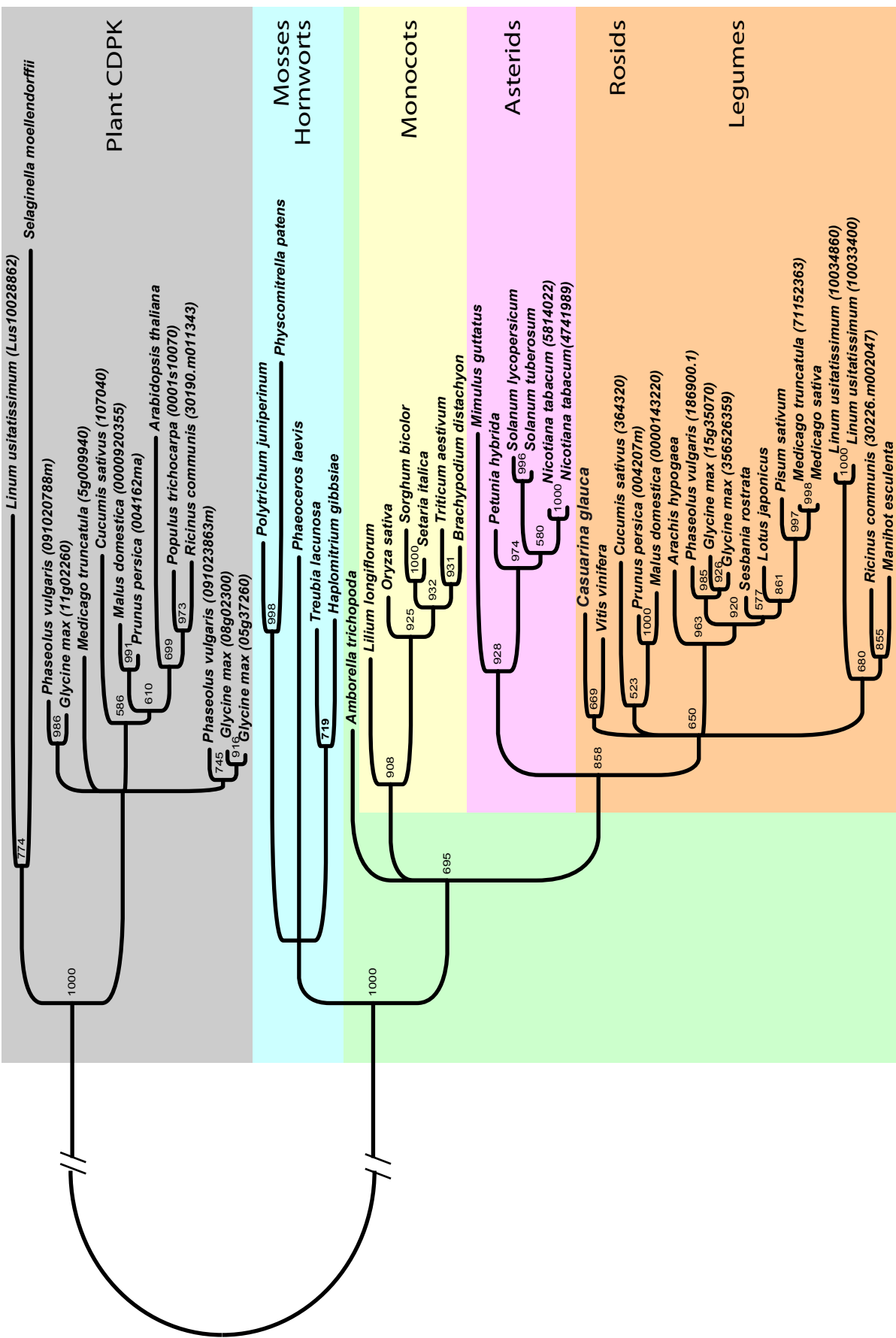

Supplement: Figure S2 — Maximum likelihood phylogeny of Calcium-Dependent Protein Kinases (CDPKs) similar to CgCCaMK. Branches with less than 50% bootstrap support were collapsed. All plant CCaMKs cluster together; other distantly related CDPK from Rosids and Selaginella were also included as an outgroup. Corresponding accession numbers are listed in Supporting Information section. (PDF) [file pone.0064515.s002.pdf]

**Figure S3**

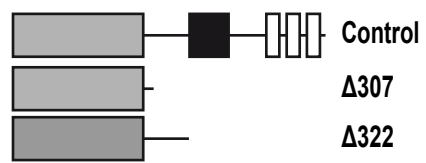

Supplement: Figure S3 — Schematic representation of truncated CgCCaMK constructs. Gray box: kinase domain; black box: calmodulin binding domain; empty box: EF hand. (PDF) [file pone.0064515.s003.pdf]
