# Supplementary material for: The Independent Acquisition of Plant Root Nitrogen-Fixing Symbiosis in Fabids Recruited the Same Genetic Pathway for Nodule Organogenesis
Source: PLoS One. 2013 May 31;8(5):e64515. doi: 10.1371/journal.pone.0064515 (PMC3669324; doi:10.1371/journal.pone.0064515)
Supplement: File S1 — Primer sequences. Accession numbers. (DOC) [file pone.0064515.s004.doc]

**Supporting information**

**Primer sequences**

*Att*B1-CgCCaMKRNAiUTR-F3: *ggggacaagtttgtacaaaaaagcaggct*AGAGTGCTGGCGAAGCCATGCATG

*Att*B2-CgCCaMKRNAiUTR-R4: *ggggaccactttgtacaagaaagctgggt*CTTGAACGACTGCAAAGAGCCAAG

*Att*B1-CgCCaMKRNAiCAM-F1:

*ggggacaagtttgtacaaaaaagcaggct*ATCTTGTACATCCTGCTCTCTGGG

*Att*B2-CgCCaMKRNAiCAM-R2: *ggggaccactttgtacaagaaagctgggt*GACTTCAGCTTCTTTGTCCG

**Accession numbers**

Sequence data from this article can be found in the EMBL/GenBank or Phytozome databases under the following accession numbers:

***Casuarina glauca*** ***CCaMK*:** Sequence data were deposited in EMBL/GenBank databases under the following accession numbers: *C. glauca* genomic fragment (HF952924) and mRNA (HF952923).

**Plant CPDKs:** *Arabidopsis* *thaliana* (At4g23650), *Cucumis* *sativus* (Cucsa.107040.1), *Glycine* *max* (Glyma08g02300.1; Glyma05g37260.1; Glyma11g02260.1), *Linum* *usitatissimum* (Lus10028862), *Malus* *domestica* (Mdp0000920355), *Medicago truncatula* (Medtr5g009940), *Phaseolus* *vulgaris* (Phvulv091020788m; Phvulv091023863m), *Populus* *trichocarpa* (0001s10070), *Prunus* *persica* (Ppa004162m), *Ricinus* *communis* (30190.m011343), *Selaginella moellendorffii* (GI: 302810917).

**Moss & hornworts CCaMKs:** *Haplomitrium* *gibbsiae* (GI: 319918041), *Phaeoceros* *laevis* (GI: 319918057), *Physcomitrella* *patens* (GI: 168042670), *Polytrichum* *juniperinum* (GI: 319918049), *Treubia* *lacunosa* (GI: 319918043).

**Plant CCaMKs:** *Amborella* *trichopoda* (GI: 319918081), *Arachis* *hypogaea* (GI: 195542474), *Brachypodium* *distachyon* (GI:357128896), *Cucumis* *sativus* (Cucsa.364320), *Glycine* max (GI: 356526359; Glyma15g35070), *Lilium* *Longiflorum* (GI: 71152362), *Linum* *usitatissimum* (Lus10033400; Lus10034860), *Lotus* *japonicus* (GI: 116634228), *Malus* *domestica* (MDP0000143220), *Manihot* *esculenta* (cassava4.1_026542m), *Medicago* *sativa* (GI: 260619579), *Medicago* *truncatula* (GI: 71152363), *Mimulus guttatus* (mgv1a020107m), *Nicotiana* *tabacum* (GI: 5814022; GI: 4741989), *Oryza* *sativa* (05g0489900), *Petunia* *hybrida* (EF592572), *Phaseolus* *vulgaris* (Phvulv1.011G186900.11), *Pisum* *sativum* (GI: 71152364), *Prunus* *persica* (ppa004207m), *Ricinus* *communis* (30226.m002047), *Sesbania* *rostrata* (GI: 186909461), *Setaria* *italica* (Si021787m ), *Solanum* *lycopersicum* (01g096820.2.1), *Solanum* *tuberosum* (01g034320.1.1), *Sorghum* *bicolor* (GI: 242094177), *Triticum* *aestivum* (GI: 300488217), *Vitis* *vinifera* (GI:359487043).
